# Phylogenetic distribution of DNA topoisomerase VI and its distinction from SPO11

**DOI:** 10.1093/nargab/lqae085

**Published:** 2024-08-06

**Authors:** Adam M B Allen, Anthony Maxwell

**Affiliations:** Department of Molecular Microbiology, John Innes Centre, Norwich Research Park, Norwich NR4 7UH, UK; Department of Molecular Microbiology, John Innes Centre, Norwich Research Park, Norwich NR4 7UH, UK

## Abstract

DNA topoisomerases (topos) are major targets for antimicrobial and chemotherapeutic drugs due to their fundamental roles in regulating DNA topology. Type II topos are essential for chromosome segregation and relaxing positive DNA supercoils, and are exemplified by topo II in eukaryotes, topo IV and DNA gyrase in bacteria, and topo VI in archaea. Topo VI occurs ubiquitously in plants and sporadically in bacteria, algae, and other protists and is highly homologous to Spo11, which initiates eukaryotic homologous recombination. This homology makes the two complexes difficult to distinguish by sequence and leads to discrepancies such as the identity of the putative topo VI in malarial *Plasmodium* species. A lack of understanding of the role and distribution of topo VI outside of archaea hampers its pursuit as a potential drug target, and the present study addresses this with an up-to-date and extensive phylogenetic analysis. We show that the A and B subunits of topo VI and Spo11 can be distinguished using phylogenetics and structural modelling, and that topo VI is not present in *Plasmodium* nor other members of the phylum Apicomplexa. These findings provide insights into the evolutionary relationships between topo VI and Spo11, and their adoption alongside other type II topos.

## Introduction

The helical structure of duplex DNA has a fundamental consequence: for DNA metabolism to occur, the two intertwined strands of DNA must separate to expose their bases to the enzymatic machinery. The processes that induce local helix unwinding generate mechanical strain on the DNA that is relieved by the formation of deleterious topological states which stall this machinery. A group of enzymes called DNA topoisomerases (topos) have evolved to resolve these topological states to allow the enzymatic machinery to advance (2). Topos modify DNA topology by introducing transient breaks into the DNA and either passing a segment of DNA through the break or rotating around an uncleaved strand, and then resealing the break (2). DNA cleavage by topos involves the formation of a phosphotyrosyl linkage between an active-site tyrosine and a phosphodiester bond (2). Topos are classified according to whether they introduce transient single-strand breaks (type I) or double-strand breaks (DSBs) (type II) in DNA, thereby changing the linking number of DNA by 1 or 2, respectively, during each reaction cycle (2).

Type II topos are ATP- and Mg^2+^-dependent enzymes that can decatenate newly replicated intertwined DNA molecules and relax positively supercoiled DNA (2). Type II topos share many common structural modules, including a GHKL ATPase domain, a transducer domain, a metal-binding toprim domain, and a DNA-cleaving winged-helix domain (WHD), and are subdivided into type IIA and type IIB based on the arrangement of these modules (2). The type IIA family is typified by eukaryotic topo II, and bacterial topo IV and DNA gyrase, and these enzymes generate DSBs with four-nucleotide overhangs (2). Gyrase is also found ubiquitously in plants and sporadically in protists and archaea (3), and is unique in its ability to wrap a segment of covalently-closed double-stranded DNA around itself and introduce negative supercoils (4).

Type II topos are essential for cellular life and so it came as no surprise when one was eventually discovered in archaea in 1994 (5). What was surprising, however, was that this enzyme was shown to form a heterotetrameric complex like the bacterial type II topos but to be sensitive to several inhibitors of eukaryotic type II topos (5). This archaeal enzyme, now termed topo VI, was also shown to possess limited sequence homology to both bacterial topo IV and eukaryotic topo II and was thus classified as the first member of a new subfamily of topos: type IIB (6). Topo VI is now thought to be ubiquitous in the archaea domain of life, except in the order Thermoplasmatales (7,8), and has also been identified in plants (9,10), protists (11), and bacteria (12). The topo VI complex comprises two A subunits (topo VI-A) and two B subunits (topo VI-B) (5) and generates DSBs with two-nucleotide overhangs (13). The other member of the type IIB family is topo VIII, which is found in free and integrated plasmids in archaea and bacteria (14). Type IIB topos possess a structural module absent in the type IIA enzymes called the helix-2-turn-helix (H2TH) domain, which contributes to the preferential binding of topo VI to supercoiled DNA and the coupling of ATP hydrolysis to strand passage (15).

In topo VI, strand passage occurs via a two-gate mechanism whereby a T-segment is captured at the ATP-gate and passed through a DSB in the G segment at the DNA-gate (1,16,17). The topo VI-A dimer serves as the DNA-gate (16), and the ATP-gate is formed by the GHKL clamp in topo VI-B (1,17). ATP binding triggers topo VI-B dimerization, which captures the T-segment in the internal cavity and induces G-segment cleavage by the topo VI-A dimer (1,16,17). ATP hydrolysis and/or phosphate release then causes a conformational change that allows the T-segment to pass through the DNA gate (1,16,17). Finally, the G-segment is resealed, and the ATP gate opens to reset the enzyme for another cycle.

Topo VI-A is highly homologous to Spo11, a protein that introduces permanent DSBs to initiate homologous recombination in eukaryotes (6). Spo11 features a toprim domain and a WHD, and possesses all the key invariant residues found in topo VI-A (6). Although most eukaryotes encode a single *spo11* gene, the genomes of plants and protists can encode multiple copies (9,11). The model plant *Arabidopsis thaliana* possesses three non-redundant Spo11 paralogues (SPO11-1, SPO11-2, SPO11-3) (9,18) that are conserved in plants and some algae (9,11), but only SPO11-1 and SPO11-2 are necessary for meiotic DSB formation (19–21). *A*. *thaliana* SPO11-3 has been shown to interact with an orthologue of archaeal topo VI-B (TOP6B) to form a functional topo VI complex that participates in endoreduplication (9,10,22,23), and is therefore a canonical topo VI-A rather than a canonical Spo11. The topo VI complex in plants requires two accessory proteins, RHL1 and BIN4, which both exhibit *in vitro* DNA binding activity (24,25). RHL1 and BIN4 homologues are present in all topo VI-possessing eukaryotes but are not found in other organisms (26).

Meiotic DSB formation has been shown to require an interaction between the Spo11 dimer and a structural homologue of topo VI-B, which is called MTOPVIB (27) and TOPOVIBL (28) in plants and animals, respectively. Structural homologues of topo VI-B have also been discovered in yeast (28,29), insects (28), and protists (26), giving rise to what is now referred to as the topo VIB-like family. It seems, therefore, that meiotic DSB formation is performed by a conserved topo VI-like scaffold. Although Spo11 and topo VI-A share high sequence homology (6), topo VIB-like shares limited sequence homology with topo VI-B and possesses degenerate versions of the GHKL and transducer domains (27,28). The GHKL-like and transducer domain-like folds are diverse in topo VIB-like, with each fold from different organisms possessing various degrees of degeneracy from their structurally homologous folds in topo VI-B (26–28). The GHKL-like domain of the topo VIB-like family is particularly variable, with metazoan sequences lacking nearly all the key residues required for ATP binding and hydrolysis that are conserved in topo VI-B, and some protist sequences possessing nearly all of them (26). Topo VIB-like are mostly reported to lack a H2TH domain (26,28) and to instead possess an unconserved ‘Linker’ domain (26), however a H2TH-like domain has been reported in MTOPVIB (27). A consequence of the discovery of the topo VIB-like family is that the presence of a eukaryotic gene product with homology to topo VI-B is no longer sufficient to signify the existence of a topo VI enzyme in that species.

It has been suggested that the genome of the malarial parasite genus *Plasmodium* harbours a protein resembling topo VI-B, given its significant sequence homology with the N-terminal GHKL domain, albeit with limited homology to the C-terminal region (11,30). *Plasmodium* spp. also possess two Spo11 paralogues, one of which has been proposed to resemble topo VI-A (31). The *P. falciparum* putative topo VI-B and topo VI-A proteins have been shown to interact with each other, to complement a yeast topo II null mutant, to possess decatenation activity in a yeast cell extract, and to be recruited to the mitochondria (31,32). Furthermore, the proposed decatenation activity of these subunits was shown to be inhibited by radicicol (31), a known inhibitor of *Saccharolobus shibatae* topo VI (33). These findings suggest that *Plasmodium* spp. possess a functional topo VI that functions in the segregation of the mitochondrial genome, however, other studies raise doubts. For example, topo VI-A species have been shown to dimerize in the absence of topo VI-B (16,27), whereas the *P*. *falciparum* putative topo VI-A (31), like other canonical Spo11 species (27,34), is unable to dimerize in the absence of its B subunit partner. Also, a phylogenetic study has defined the Spo11 paralogues in *Plasmodium* spp. as orthologues of plant SPO11-1 and SPO11-2 and has proposed that SPO11-3/topo VI-A is absent in this genus (11). Furthermore, the *P*. *falciparum* putative topo VI-A has been shown to complement the sporulation defect of a yeast Spo11 null mutant (32). Overall, these alternative findings suggest that the putative topo VI subunits in *Plasmodium* spp. instead form a meiotic topo VI-like complex.

Establishing the evolution of topo VI in the tree of life is necessary to reveal insights into chromosome segregation and homologous recombination, and to explore the enzyme's potential as an herbicide and drug target. Here, an extensive phylogenetic analysis was performed to fully characterize the topo VI and topo VI-like subunits from bacteria, archaea and eukaryotes. This work has three aims: to provide a means of distinguishing the subunits of the topo VI and topo VI-like complex, to describe the distribution of topo VI in bacteria and protists, and to determine whether *Plasmodium* spp. possess topo VI.

## Materials and methods

### Collection of topo VI and Spo11 sequences

Protein sequences were obtained using the online National Center for Biotechnology Information (NCBI) protein database tool (https://www.ncbi.nlm.nih.gov/protein), by searching for sequences annotated with DNA topoisomerase (topo) VI, type II topo or Spo11 identifiers. Unannotated and misannotated homologues were identified by employing BLASTp (35) and PSI-BLAST (36) searches on the NCBI web server and HMMER3 analysis (37) on the European Molecular Biology Laboratory (EMBL) HMMER web server (38), using known topo VI/Spo11 sequences as queries. Sequences obtained from metagenome-assembled genomes were discarded, except for Asgard archaeal sequences, and protist topo VIB-like sequences were taken from the supplementary data of Brinkmeier *et al.* (26). Pooled topo VI-A/Spo11 sequences were subject to a preliminary alignment, and only sequences that contained the conserved catalytic tyrosine residue (Y106 in *Methanosarcina mazei* topo VI-A) were retained. Enzyme host organisms were subjected to taxonomic classification using the NCBI Taxonomy Browser (39), and taxonomy tables were created in Microsoft Excel.

### Phylogenetic analysis and structural modelling

Sequences were aligned using the EMBL-EBI Multiple Sequence Alignment tool (40) and the T-Coffee algorithm (41) and were trimmed using trimAI (42). Maximum-likelihood phylogenetic trees were constructed using the IQ-TREE algorithm on XSEDE (43) on the Cipres Science Gateway web server (44), and the ultrafast bootstrap approximation approach with 1000 bootstrap replicates (45) was used to estimate the phylogenetic support of clades. Unrooted phylogenetic trees were visualized using the Interactive Tree Of Life (iTOL) web-based tool (46) and pruned phylogenetic trees were generated using the online phyloT tool (https://phylot.biobyte.de). The ESPript 3.0 programme (47) was used to render sequence similarities from the aligned amino acid sequences and pairwise sequence identity calculations were performed using the online Sequence Manipulation Suite (48). Protein structures were modelled using the AlphaFold2 algorithm (49) and ColabFold software (50) and were visualized using the CCP4 Molecular Graphics (CCP4MG) program suite (51). Figures were created with BioRender.com.

## Results

### Collection of topo VI and Spo11 sequences

To determine whether the homologous subunits of the Spo11 and topo VI complexes can be distinguished using phylogenetic analysis, sequences were collected from the NCBI protein database and from BlastP, PSI-BLAST and HHMER analyses. To capture a reliable representation of the full evolutionary landscape of these proteins, a dataset was acquired that encompassed a diverse range of taxonomic classes, with each sequence being associated with a unique family and, in many cases, a unique order (Supplementary Tables S1–S3). A total of 223 topo VI-A/Spo11 sequences and 108 topo VI-B sequences were collected from the three domains of life, with each topo VI-B-possessing organism retaining its corresponding topo VI-A subunit in the collection. Due to the uncertain identity of the putative topo VI subunits in *Plasmodium* spp., it was necessary to include homologues from other Apicomplexa species in the dataset to assist with their elucidation. *Plasmodium* spp. possess two SPO11 paralogues and one putative TOP6B (pTOP6B), and these subunits are conserved in Apicomplexa, with representatives identified here in four orders across two classes (Supplementary Table S4). Seven pTOP6B sequences from Apicomplexa were therefore added to the topo VI-B collection. It has previously been reported that all sequenced archaea genomes possess topo VI, except those in the order Thermoplasmatales where the enzyme has been replaced by gyrase (7). In the present study, topo VI genes have been identified in two Thermoplasmatales archaeal species, namely, *Cuniculiplasma divulgatum* and *Thermogymnomonas acidicola*.

### Topo VI-A and Spo11 can be distinguished by phylogenetic analysis

A series of key conserved residues present in the topo VI-A/Spo11 WHD and toprim domain have been previously characterized by biochemical and genetic analyses (6,52–56), and these are present in all members of the topo VI-A/Spo11 family (Supplementary Figure S1). The only exception is that the second aspartate residue of the metal-binding DxD motif is always substituted for asparagine in SPO11-2 from plants, and sometimes also in SPO11-2 from protists (Supplementary Figure S1). Given the lack of an identifiable motif exclusive to either topo VI-A or Spo11, it is necessary to analyse their phylogenies to distinguish them at the sequence level. A previous study was able to separate SPO11-1, SPO11-2, SPO11-3, and prokaryotic topo VI-A using phylogenetic analysis (11), but did so without significant evolutionary diversity in their dataset. For example, limited bacterial and eukaryotic topo VI-A sequences were included, and many sequences clustered in the less inclusive taxonomic levels. Moreover, this research employed rooted phylogenetic trees, which are less effective for clustering homologous sequences compared to unrooted trees (57).

**Figure 1.**
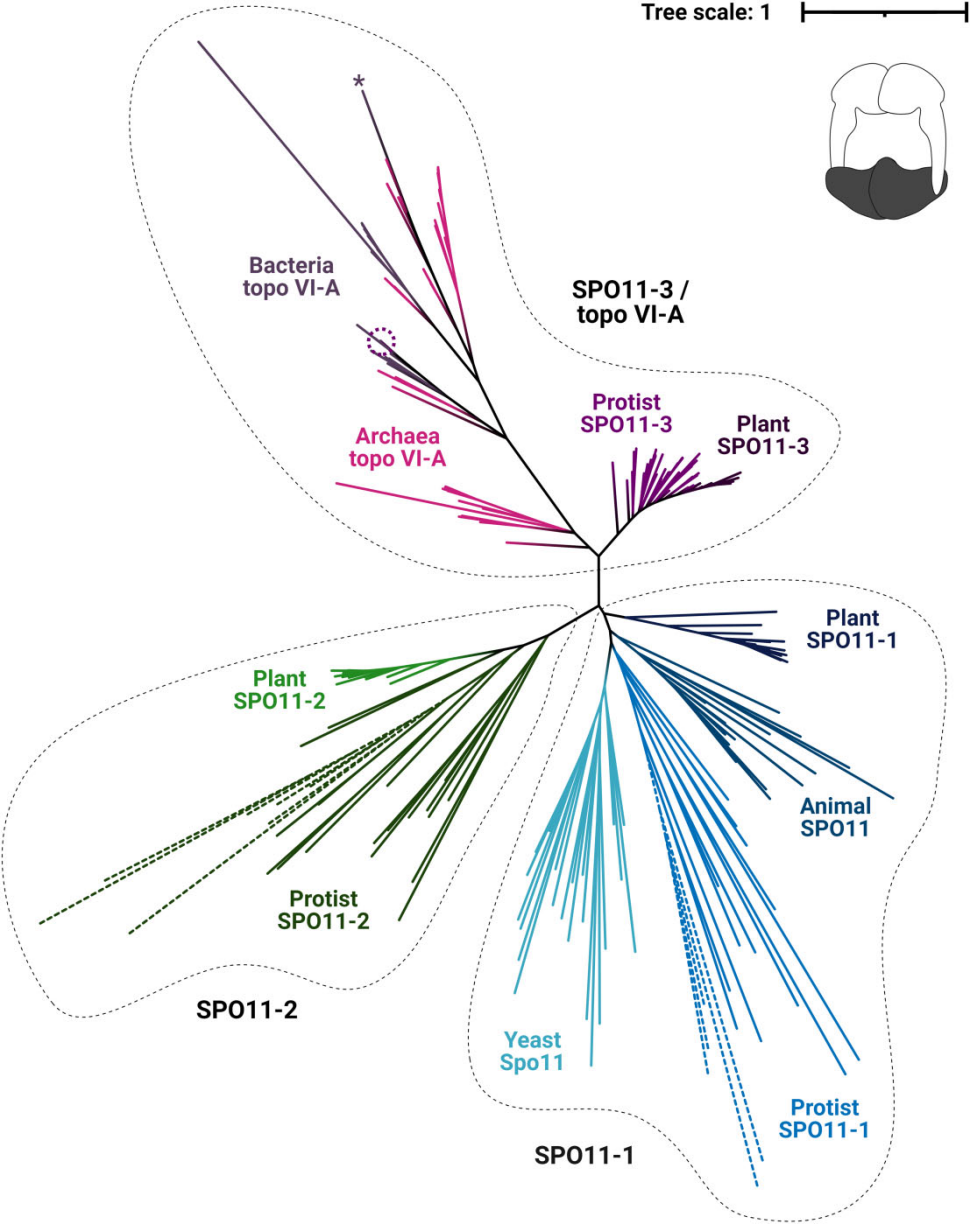
Phylogenetic tree of the topo VI-A/Spo11 family: An unrooted maximum-likelihood tree using 223 aligned topo VI-A/Spo11 sequences including 33 archaea, 17 bacteria, and 173 eukaryotes. The topo VI-A/Spo11 family splits into three major clades: topo VI-A/SPO11-3, SPO11-1, and SPO11-2. The dotted leaves represent Apicomplexa SPO11 paralogues, the dotted circle highlights the protist *Cladocopium goreaui* (topo VI-A leaf), and * highlights *Ferrimicrobium acidiphilum* (topo VI-A leaf). Tree scale represents the number of amino acid substitutions per site and the cartoon in the top right corner depicts the structure of topo VI (1) with the A subunit shaded in black. Tree was generated from a trimmed alignment possessing 263 amino acids.

To determine the phylogenetic relationships within the topo VI-A/Spo11 family, a diverse and extensive dataset of sequences was curated using updated genetic resources and subject to phylogenetic tree analysis. The topo VI-A/Spo11 family splits into three major clades on an unrooted phylogenetic tree: topo VI-A/SPO11-3, SPO11-2 and SPO11-1 (Figure 1 and Supplementary Figure S2). Topo VI-A/SPO11-3 is present only in archaea, bacteria, plants, and protists; SPO11-1 is present only in yeast, protists, and multicellular eukaryotes; and SPO11-2 is present only in plants and protists (Figure 1). Topo VI-A/SPO11-3 was highly likely to have been present in the last common ancestor (LCA) of archaea and eukaryotes, given that at least one of the homologues is conserved in both domains, and is likely to have undergone two gene duplication events in eukaryotes to give rise to SPO11-1 and SPO11-2 (Figure 1). SPO11-1 exhibits a widespread distribution in eukaryotes, whereas the distribution of SPO11-2 is much narrower This suggests that SPO11-3 and SPO11-1 were separated first, likely in the last eukaryotic common ancestor (LECA), and that SPO11-1 and SPO11-2 were separated second, likely in an early modern eukaryote after the LECA. Bacterial topo VI-A separates into two clusters within the archaeal topo VI-A clade, suggesting that archaeal topo VI-A was transferred to bacteria at least two times via horizontal gene transfer (HGT) and was preceded by multiple secondary transfers (Figure 1). One of these clusters lies close to Euryarchaeota archaea of the order Methanococcales, and the second cluster lies close to a few species of DPANN and Euryarchaeota archaea. Topo VI-A from the bacterium *Ferrimicrobium acidiphilum* sits outside of the bacterial clusters on its own and is adjacent to a few TACK archaea, suggesting a possible third transfer (Figure 1).

An anomaly in the prokaryotic topo VI-A clade is the position of the eukaryote *Cladocopium goreaui* within the bacterial Pirellulales clade (Figure 1). *C*. *goreaui* is a dinoflagellate member of the superphylum Alveolata that inhabits marine ecosystems alongside Pirellulales bacteria. Topo VI-A from *C*. *goreaui* exhibits only 18.0% sequence identity with SPO11-3 from fellow dinoflagellate *Prorocentrum donghaiense* but 55.4% identity with topo VI-A from the Pirellulales bacterium *Thermogutta terrifontis*. Dinoflagellates have been shown to possess multiple genes from various bacterial sources (58–60) and it is likely that *C*. *goreaui* acquired topo VI-A from a close relative of Pirellulales by HGT. SPO11-3 from *P*. *donghaiense*, however, sits alongside SPO11-3 from other protists, such as Stramenopila and Rhizaria (Figure 1). Along with Alveolata, these phyla collectively constitute the supergroup SAR, which has diverged from the superphyla Cryptista, Haptista and Archaeplastida, the latter of which contains red algae, green algae, and plants (61). As expected, SAR SPO11-3 sequences all cluster closely to SPO11-3 from Cryptista, Haptista, and Archaeplastida, whereas the two SPO11 paralogues of Apicomplexa, which are members of Alveolata, are present only in the SPO11-1 and SPO11-2 clades (Figure 1). Therefore, Apicomplexa, unlike their close dinoflagellate and SAR relatives, do not possess SPO11-3.

### The GHKL domain is highly conserved In topo VI-B

**Figure 2.**
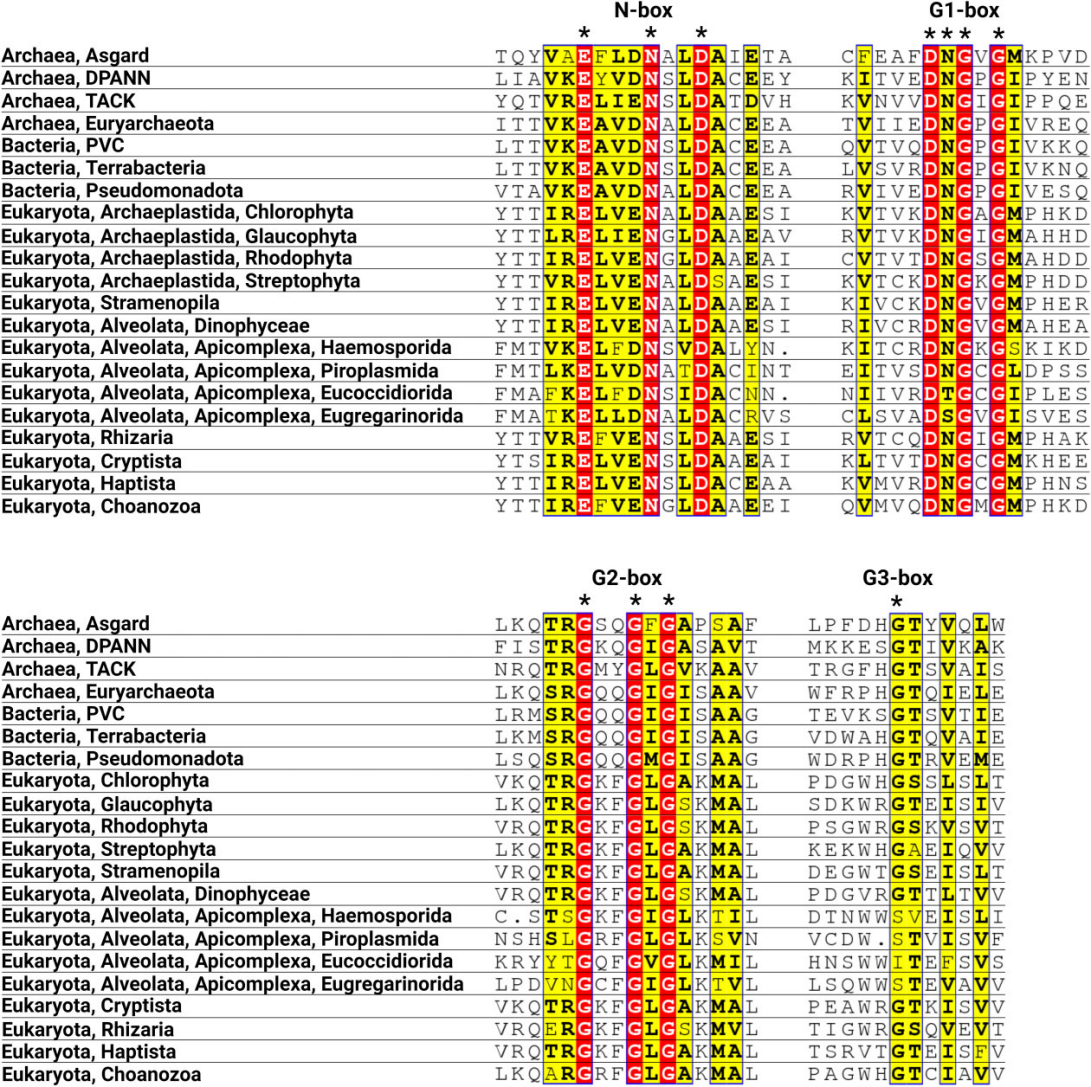
Sequence alignment of the topo VI-B GHKL domain: Canonical topo VI-B members contain key invariant ATP-binding residues in the GHKL domain (N-, G1-, G2, G3-box). The putative TOP6B subunits from Apicomplexa lack the G3-box glycine. Topo VI-B sequences are represented by Asgard (*Candidatus Odinarchaeum yellowstonii*), DPANN (*Candidatus Nanobsidianus stetteri*), TACK (*Saccharolobus shibatae*), Euryarchaeota (*Methanosarcina mazei*), PVC (*Pirellula staleyi*), Terrabacteria (*Cyanobacterium TDX16*), Pseudomonadota (*Bradyrhizobium sediminis*), Chlorophyta (*Chlamydomonas reinhardtii*), Glaucophyta (*Cyanophora paradoxa*), Rhodophyta (*Chondrus crispus*), Streptophyta (*Arabidopsis thaliana*), Stramenopila (*Pythium insidiosum*), Dinophyceae (*Prorocentrum donghaiense*), Rhizaria (*Lotharella oceanica*), Cryptista (*Guillardia theta*), Haptista (*Phaeocystis globosa*), and Choanozoa (*Monosiga brevicollis*). pTOP6B sequences are represented by Haemosporida (*Plasmodium falciparum*), Piroplasmida (*Babesia microti*), Eucoccidiorida (*Cryptosporidium ubiquitum*), and Eugregarinorida (*Porospora cf. gigantea B*).

Topo VI-A and Spo11 possess a high degree of sequence homology, however, the topo VI-B and topo VIB-like families have been shown to exhibit differential sequence homology in their GHKL and GHKL-like folds (27,28). The GHKL domain is an ATP-binding module characterized by four invariant Bergerat motifs: N-box, G1-box, G2-box and G3-box (6,62), and topo VIB-like subunits have been shown to possess limited sequence homology in the G1-box and G2-box regions and to completely lack an N-box. To determine the conservation of these residues in the topo VI-B dataset, a sequence alignment of the GHKL domain was performed. All topo VI-B sequences from prokaryotes and eukaryotes possess all the key ATP-binding residues in the N-box, G1-box, G2-box, and G3-box (Figure 2). The putative GHKL domain of Apicomplexa pTOP6B is highly homologous to the topo VI-B GHKL domain and possesses all the Bergerat motifs except the G3-box (Figure 2). The absence of a G3-box may not be sufficient to elucidate the identity of Apicomplexa pTOP6B, but this feature makes the subunit distinct from TOP6B from other SAR organisms, where the GHKL is fully conserved.

### The Apicomplexa putative TOP6B is phylogenetically distinct from eukaryotic TOP6B

**Figure 3.**
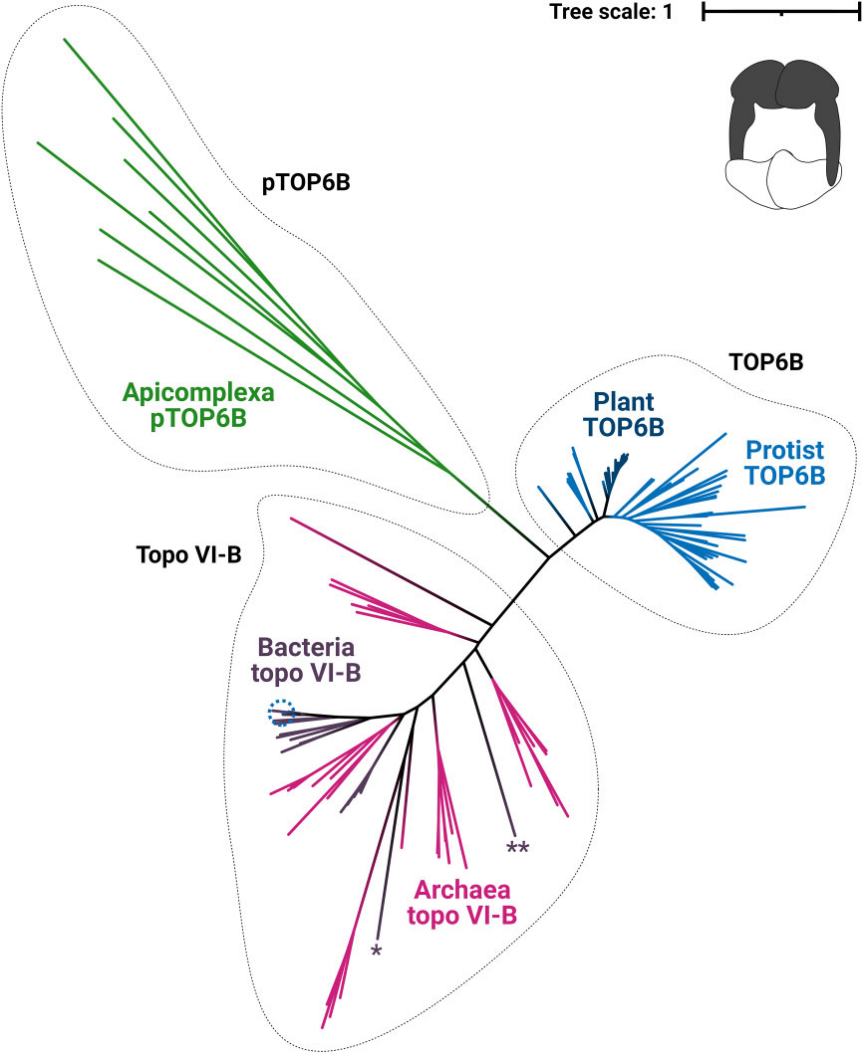
Phylogenetic tree of the topo VI-B family: An unrooted maximum-likelihood tree using 108 topo VI-B sequences, including 33 archaea, 17 bacteria, and 58 eukaryotes, aligned with 7 putative TOP6B (pTOP6B) sequences from Apicomplexa. The topo VI-B family splits into two clades: prokaryotic topo VI-B and eukaryotic TOP6B. The putative TOP6B (pTOP6B) from Apicomplexa form a separate clade. The dotted circle highlights the protist *Cladocopium goreaui* (topo VI-B leaf), * highlights *Ferrimicrobium acidiphilum* (topo VI-B leaf), and ** highlights the anomalous *Bdellovibrio bacteriovorus* (topo VI-B leaf). Tree scale represents the number of amino acid substitutions per site and the cartoon in the top right corner depicts the structure of topo VI (1) with the B subunit shaded in black. Tree was generated from a trimmed sequence alignment possessing 458 amino acids.

To determine the identity of Apicomplexa pTOP6B, a phylogenetic analysis was performed on the topo VI-B dataset. Topo VIB-like subunits lack sequence homology to topo VI-B, and so were not included in the analysis. A phylogenetic tree of the topo VI-B family splits into two clades: prokaryotic topo VI-B and eukaryotic TOP6B (Figure 3). Apicomplexa pTOP6B form a separate clade that is distantly related to TOP6B from SAR organisms and other eukaryotes, suggesting that the subunit is not a member of the topo VI-B family.

Bacterial topo VI-B separates into two clusters adjacent to Euryarchaeota within the archaeal topo VI-B clade, suggesting that archaeal topo VI-B, like archaeal topo VI-A, was transferred to bacteria at least two times via HGT (Figure 3). Topo VI-B from the bacterium *F*. *acidiphilum*, like its corresponding topo VI-A, sits outside of the bacterial clusters on its own and is distant from other archaeal topo VI-B sequences. This supports a third transfer archaeal topo VI to bacteria. Topo VI-B from *Bdellovibrio bacteriovorus*, unlike its corresponding topo VI-A, also sits outside of the two bacterial clusters (Figure 3). Given the larger dataset used for the topo VI-A/Spo11 sequence alignment, and the presence of the large pTOP6B outgroup in the topo VI-B alignment, the placement of *B*. *bacteriovorus* topo VI-B is likely to be anomalous. The unusual position of topo VI-A from the eukaryote *C*. *goreaui* within the bacterial Pirellulales clade is reproduced by its corresponding partner in the topo VI-B tree (Figure 3). *C*. *goreaui* topo VI-B exhibits only 19.2% sequence identity with TOP6B from fellow dinoflagellate *P*. *donghaiense* but exhibits 62.0% identity with topo VI-B from the Pirellulales bacterium *T*. *terrifontis*. It is therefore likely that *C*. *goreaui* acquired both topo VI subunits from a close relative of Pirellulales by HGT.

### Topo VI-B and topo VIB-like can be distinguished by structural modelling

**Figure 4.**
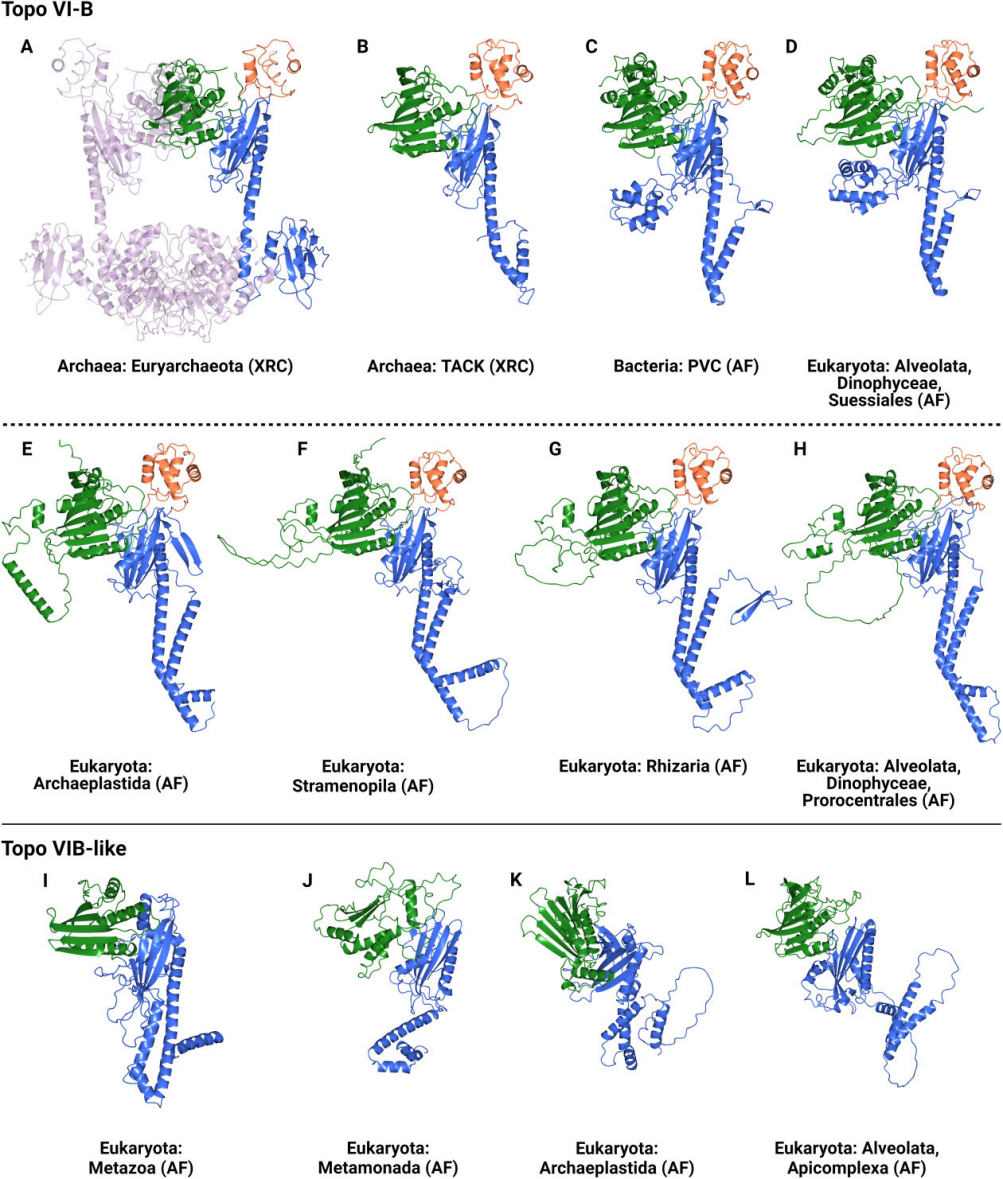
Topo VI-B and topo VIB-like structural modelling: The X-ray crystal (XRC) structure of (**A**) *Methanosarcina mazei* (Euryarchaeota) topo VI (PDB code: 2Q2E) is a heterotetramer formed of two topo VI-As and two topo VI-Bs. The *M*. *mazei* topo VI-B structure is comparable to (**B**) the *Saccharolobus shibatae* (TACK) topo VI-B XRC structure (PDB code: 2ZBK), which both feature a transducer domain (blue), a H2TH domain (orange), and a GHKL domain (green). AlphaFold (AF) structural modelling predicts that the topo VI-B structure is also conserved in (**C**) PVC (*Pirellula staleyi*) bacteria, and (**D**) Archaeplastida (*Arabidopsis thaliana*), (**E**) Stramenopila (*Pythium insidiosum*), (**F**) Rhizaria (*Lotharella oceanica*) and (**G**) Prorocentrales (*Prorocentrum donghaiense*) eukaryotes. Topo VI-B from the eukaryote (**H**) *Cladocopium goreaui* (Suessiales) was acquired from a PVC bacterium by horizontal gene transfer and shares a common alpha-helical bundle in its transducer domain. The topo VIB-like family structures possess a transducer-like domain (blue) and a GHKL-like domain (green), but do not possess a H2TH domain, and are represented by (**I**) Metazoa (*Homo sapiens*), (**J**) Metamonada (*Giardia intestinalis*), (**K**) Archaeplastida (*A*. *thaliana*), and (**L**) Apicomplexa (*Plasmodium falciparum*).

Topo VI-A and Spo11 can be distinguished by phylogenetic analysis, however, topo VI-B and topo VIB-like exhibit limited sequence homology and so are unsuitable for sequence alignment. Therefore, AlphaFold (49) modelling was employed to investigate whether the two subunits can be distinguished from their predicted structures. The crystal structure of the topo VI heterotetramer has been solved for both *Methanosarcina mazei* (1) (PDB code: 2Q2E) and *Saccharolobus shibatae* (63) (PDB code: 2ZBK). These structures feature a H2TH domain, an ATPase GHKL domain, and a transducer domain that interacts with topo VI-A. Structural modelling suggests that these three folds, and the overall subunit shape, are conserved in topo VI-B from archaea, bacteria, and eukaryotes (Figure 4 and Supplementary Figures S2 and S3). *M. mazei* topo VI-B possesses a C-terminal domain (CTD) adjacent to its transducer domain that adopts an immunoglobulin-like fold (1), and a recent study has shown, using AlphaFold, that this C-terminal extension is unique to archaeal species that possess a gyrase, hence its absence in *S. shibatae* topo VI (8). AlphaFold predictions show that topo VI-B from all bacterial and eukaryotic species tested do not possess an analogous immunoglobulin-like fold (Figure 4 and Supplementary Figures S2 and S3).

The transducer domain models of topo VI-B feature a long α-helix (68 Å in *M. mazei*, 88 Å in *A*. *thaliana*, 75 Å in *Bradyrhizobium sediminis*) that spans the internal cavity of the enzyme and interacts with topo VI-A (Figure 4 and Supplementary Figures S2 and S3). In prokaryotes, this long helix is adjacent to a second shorter helix that runs back parallel to the long helix. In eukaryotes, the long helix forms a large kink and is followed by a second and third helix that together form a V-shape that runs adjacent to the long helix and mimics the kink. Topo VI-B from the choanozoan *Monosiga brevicollis* is unique in that its long transducing α-helix is approximately half the length of those from other topo VI-B proteins (48 Å) (Supplementary Figure S4), significantly reducing the size of the central cavity of topo VI from this organism. Eukaryotic topo VI-B also features an additional α-helical region adjacent to the GHKL domain, which is particularly prominent in Streptophyta, Chlorophyta, Rhodophyta, and Haptista, that may interact with the opposing GHKL domain of the TOP6B dimer when the ATP-gate is closed.

Structural modelling predicts that topo VI-B from archaea and bacteria possess highly homologous folds (Figure 4 and Supplementary Figure S3), as expected for recently transferred gene products. The Pirellulales bacterium *Pirellula staleyi* possesses a unique alpha-helical bundle adjacent to the transducer domain that protrudes into the central cavity of the enzyme that is not found in other prokaryotic or eukaryotic topo VI enzymes except *C*. *goreaui*. TOP6B from *C*. *goreaui* also lacks the conserved structural features of other eukaryotic TOP6B and, given its phylogenetic placement, is therefore likely to have been acquired laterally from a Pirellulales bacterium.

Pre-AlphaFold structural modelling has proposed that MTOPVIB from plants possess a H2TH-like domain (27), but that TOPOVIBL from vertebrates and invertebrates lack this domain completely (28). Here, all members of the topo VIB-like family possess GHKL-like and transducer-like folds and can be characterized by the complete absence of a H2TH domain (Figure 4 and Supplementary Figure S5). The H2TH domain is therefore unique to topo VI-B subunits, and its role in DNA-binding is not required for the meiotic DSB machinery. In the plant and protist topo VIB-like structures, the transducer-like domains feature a small bundle of alpha-helices in place of the long transducing α-helix seen in topo VI-B (Figure 4 and Supplementary Figure S5). The human TOPOVIBL structure, however, has retained the long transducing α-helix (67 Å) and possesses a fold that more closely resembles a topo VI-B transducer domain than a topo VIB-like transducer-like domain.

Topo VI-B and topo VIB-like can be distinguished by structural modelling, and therefore AlphaFold was employed to elucidate the identity of Apicomplexa pTOP6B. The predicted structures of Apicomplexa pTOP6B lack a H2TH domain and possess a transducer domain-like fold where the long transducing α-helix is replaced with a small bundle of α-helices (Figure 4 and Supplementary Figure S5). The pTOP6B subunit from Apicomplexa is therefore unlikely to be a TOP6B and is likely instead to be a member of the topo VIB-like family. Given that the phylogenetic and structural data suggest that Apicomplexa do not possess SPO11-3 or TOP6B, it can be concluded that the phylum does not possess topo VI, contrary to previous reports (31,32). The GHKL-like domains of the topo VIB-like family have become degenerate versions of the topo VI-B GHKL folds to various degrees. For example, the GHKL-like folds of SAR topo VIB-like subunits closely resemble a canonical topo VI-B GHKL fold, whereas the GHKL-like folds from discoban and metamonad topo VIB-like structures have diverged considerably (Figure 4 and Supplementary Figure S5).

### Distribution of topo VI and its accessory proteins in the supergroup SAR

**Figure 5.**
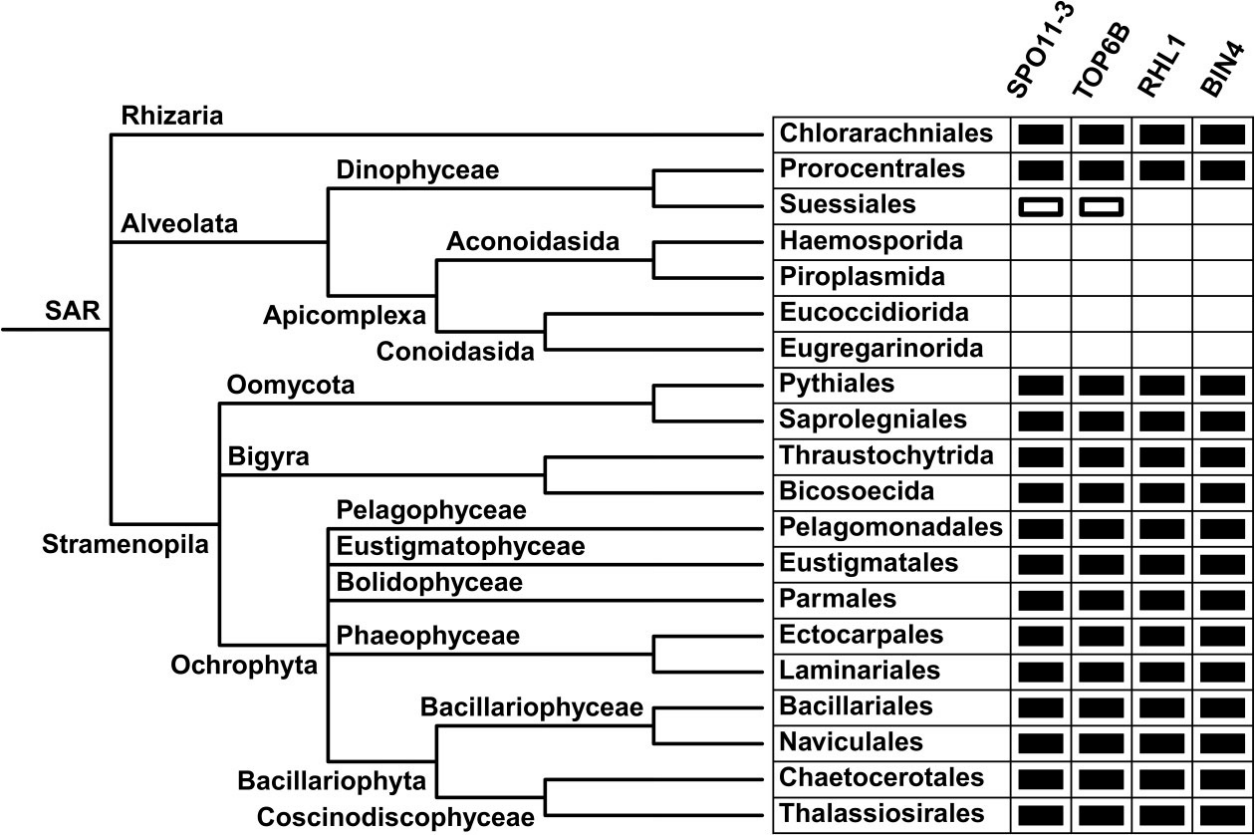
Distribution of topo VI and its accessory proteins in the SAR supergroup: RHL1 and BIN4 are always found in topo VI-possessing eukaryotes, except in *Cladocopium goreaui* (Suessiales), which obtained topo VI-A (SPO11-3) and topo VI-B (TOP6B) from bacteria via horizontal gene transfer (indicated by closed rectangles). RHL1 and BIN4 are not found in archaea, bacteria, or eukaryotes that do not possess topo VI. The absence of topo VI, RHL1, and BIN4 has been confirmed in Apicomplexa. SAR organisms are represented by Chlorarachniales (*Lotharella oceanica*), Prorocentrales (*Prorocentrum donghaiense*), Suessiales (*C*. *goreaui*), Haemosporida (*Plasmodium falciparum*), Piroplasmida (*Babesia microti*), Eucoccidiorida (*Cryptosporidium ubiquitum*), Eugregarinorida (*Porospora cf. gigantea B*), Pythiales (*Pythium insidiosum*), Saprolegniales (*Aphanomyces astaci*), Thraustochytrida (*Hondaea fermentalgiana*), Bicosoecida (*Cafeteria roenbergensis*), Pelagomonadales (*Pelagomonas calceolate*), Eustigmatales (*Nannochloropsis gaditana*), Parmales (*Tetraparma gracilis*), Ectocarpales (*Ectocarpus siliculosus*), Laminariales (*Saccharina japonica*), Bacillariales (*Fragilariopsis cylindrus*), Naviculales (*Mayamaea pseudoterrestris*), Chaetocerotales (*Chaetoceros tenuissimus*), and Thalassiosirales (*Thalassiosira pseudonana*). Branch lengths do not correspond to any scale.

There are contradictory claims as to the presence of topo VI in Apicomplexa (11,31) and the enzyme has been shown to occur only partially in Rhizaria, Alveolata and the phylum Oomycota (26). Therefore, it is necessary to confirm exactly where SPO11-3, TOP6B, and their accessory proteins, RHL1 and BIN4, are present in the supergroup SAR. In agreement with previous observations (26), the presence of SPO11-3 and TOP6B in SAR organisms always corresponds with the presence of RHL1 and BIN4, including in the dinoflagellate order Prorocentrales (Figure 5). An exception to this is that the bacterially-derived-topo VI-possessing suessiacean dinoflagellate *C*. *goreaui* does not seem to possess RHL1 or BIN4, although this organism does not strictly possess SPO11-3 nor TOP6B. RHL1 and BIN4 also seem to be missing in Apicomplexa, which supports the absence of topo VI in this phylum. The presence of topo VI is confirmed in at least 16 SAR orders and the enzyme is most abundant in Stramenopila (Figure 5).

### The presence of topo VI in bacteria is not necessarily associated with the absence of topo IV

**Figure 6.**
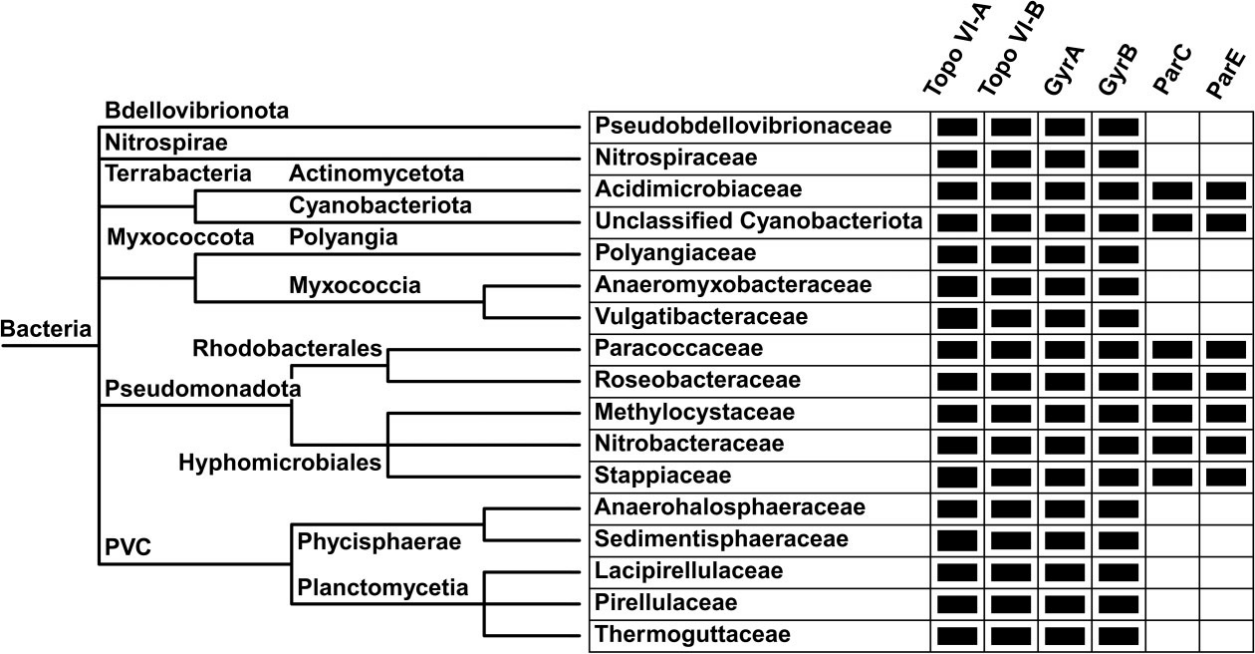
Distribution of gyrase and topo IV subunits in topo VI-possessing bacteria: Topo VI-A and topo VI-B are present in at least 17 bacterial families, all of which possess both gyrase subunits (GyrA, GyrB). Topo IV subunits (ParC, ParE) are lacking in all these families, except those in the Terrabacteria and Pseudomonadota superphyla. Topo VI-possessing bacteria are represented by Pseudobdellovibrionaceae (*Bdellovibrio bacteriovorus*), Nitrospiraceae (*Nitrospira moscoviensis*), Acidimicrobiaceae (*Ferrimicrobium acidiphilum*), Cyanobacteriota (*Cyanobacterium TDX16*), Polyangiaceae (*Pajaroellobacter abortibovis*), Anaeromyxobacteraceae (*Anaeromyxobacter dehalogenans*), Vulgatibacteraceae (*Vulgatibacter incomptus*), Paracoccaceae (*Marimonas lutisalis*), Roseobacteraceae (*Actibacterium sp. MT2.3–13A*), Methylocystaceae (*Methylocystis sp. NLS-7*), Nitrobacteraceae (*Bradyrhizobium sediminis*), Stappiaceae (*Roseibium aggregatum*), Anaerohalosphaeraceae (*Anaerohalosphaera lusitana*), Sedimentisphaeraceae (*Sedimentisphaera cyanobacteriorum*), Lacipirellulaceae (*Lacipirellula parvula*), Pirellulaceae (*Pirellula staleyi*), and Thermoguttaceae (*Thermogutta terrifontis*). Branch lengths do not correspond to any scale.

Topo VI has previously been discovered in six bacterial families that span four taxonomic classes and four phyla (12,64). In the present study, topo VI has been discovered in an additional 11 bacterial families that span an additional four classes and three phyla (Figure 6 and Supplementary Table S2). All topo VI-possessing bacteria identified so far are gram-negative, except for the gram-positive actinomycete *F*. *acidiphilum* identified here. To investigate the role of topo VI in bacteria, the complete complement of type II topos in topo VI-possessing bacteria was determined. The two gyrase subunits, GyrA and GyrB, have been found in all sequenced bacterial genomes, whereas the two homologous topo IV subunits, ParC and ParE, respectively, are present in most, but not all, bacteria (12). An exception is the bacteria *Aquifex aeolicus*, which possess a chimeric type IIA topo formed of GyrB and ParC (65). Using the sequences of *Escherichia coli* GyrA and ParC as queries for protein-protein BLAST searches, putative GyrA/ParC sequences from 17 topo VI-possessing bacteria from 17 unique taxonomic families were collected. To elucidate their identity, these sequences were aligned and classified by the presence of a GyrA-box, which is unique to GyrA and essential for the supercoiling activity of gyrase (66,67). As expected, all topo VI-possessing bacteria possessed a GyrA, but only seven possessed a ParC in addition to GyrA (Supplementary Figure S6). Protein-protein BLAST searches were then employed to confirm that each of the classified GyrA/ParC subunits were present alongside their complementary GyrB/ParE partners. Therefore, seven bacterial species from seven families are shown here to possess gyrase, topo IV, and topo VI (Figure 6), and are the first identified prokaryotes to possess a total of three type II topos. Bacterial species that possess gyrase, topo IV, and topo VI are found exclusively in the phylum Pseudomonadota and the superphylum Terrabacteria, with topo VI-possessing bacteria found in other groups lacking topo IV (Figure 6).

## Discussion

By employing phylogenetic analysis and structural modelling, the highly homologous topo VI and topo VI-like complexes can be distinguished. These methods can track the gene duplication events separating Spo11 from topo VI-A, and identify the degeneration of the GHKL, H2TH, and transducer domains that separates topo VIB-like from topo VI-B. With this knowledge, it seems likely that the malarial parasite genus *Plasmodium*, as well as other members of the phylum Apicomplexa, do not possess topo VI. This result contradicts a previous study that combined biochemical and genetic analyses to demonstrate that the *P*. *falciparum* putative topo VI subunits form an active decatenase (31). It is likely that the misinterpretation of the presence of topo VI in *Plasmodium* spp. originates from the genus possessing a topo VIB-like subunit with a GHKL-like domain that, unlike other GHKL-like domains, shares sufficient homology to the topo VI GHKL domain to be picked out in topo VI-B sequence database searches and falsely annotated as topo VI-B by automatic classification algorithms. A series of genome-wide knockout and transcriptomics studies have demonstrated that SPO11-1 and topo VIB-like from *Plasmodium* spp. have similar expression profiles *in vivo* and that these genes are upregulated in the sexual developmental stages of the parasite's life cycle (68–70), suggesting that they are likely to form a complex. SPO11-2 from *Plasmodium* spp., however, unlike SPO11-1 and topo VIB-like, has been shown to be essential in the asexual developmental stages (68,70), suggesting it performs a distinct role. These studies compound evidence for the absence of topo VI in *Plasmodium* spp., and imply that the topo VI-like complex in this genus may not comprise a dissymmetric SPO11-1/SPO11-2 heterodimer, as it does in plants (20,21), and that SPO11-2 may perform a novel and meiosis-independent function in this genus.

The SPO11-2 paralogue from *A*. *thaliana* possesses a variation of the DxD metal binding motif in which the second aspartate residue is substituted for asparagine (18). This is unusual, given that the DxD motif is highly conserved in all toprim-domain-possessing enzymes, including topoisomerases, nucleases, and primases (71), and that mutating the second DxD aspartate in *E. coli* topo I drastically reduces DNA binding (54). This alternative DxN motif is shown here to be conserved in plant SPO11-2, to be present sometimes in protist SPO11-2, but to be absent in other Spo11 paralogues. Interestingly, some protists do not possess SPO11-1, and so must therefore introduce meiotic DSBs with a topo VI-like complex comprising a SPO11-2 dimer. Green algae of the order Mamiellales, for example, lack SPO11-1 but possess a SPO11-2 with a DxN motif, which suggests that one or two DxD to DxN substitutions in the topo VI-like complex can be tolerated without impairing meiotic DSB formation. It is unclear what the effect of this substitution is on metal binding and DNA cleavage or why it is unique to the topo VI-like complex.

Cellular life requires at least one type II topo to remove the positive supercoils that form ahead of the replication and transcription machinery and to decatenate sister chromatids during chromosome segregation. Topo II, topo IV, and topo VI are highly specialized at performing these reactions, however gyrase is primarily involved in the introduction of negative supercoils into the genome. Therefore, although gyrase is essential in bacteria, most bacteria also possess topo IV to help alleviate the topological burden of nucleic acid metabolism. Topo IV is only found in bacteria and topo II is only found in eukaryotes, whereas gyrase and topo VI are widespread across all three domains of life. Gyrase-possessing archaea have been shown to maintain a negatively-supercoiled genome (72,73) and eukaryotic gyrase has been shown to complement *E*. *coli* strains carrying temperature-sensitive *gyrA* and *gyrB* mutations (74), suggesting that the unique negative supercoiling activity of bacterial gyrase is retained in these domains. Unlike gyrase, the presence of topo VI in the three domains is puzzling. The presence of topo VI in bacteria that lack topo IV can be rationalized by the requirement for a specialized decatenase (75) that participates in chromosome segregation, that is often otherwise fulfilled by topo IV. In support of the idea that topo VI in bacteria can compensate for a lack of topo IV, we have found that it is possible to complement a *parE* temperature-sensitive mutant of *E*. *coli* with topo VI from *M. mazei* (unpublished data). However, some Pseudomonadota and Terrabacteria bacteria possess topo VI in addition to topo IV and some eukaryotes possess topo VI in addition to topo II, giving rise to an apparent type II topo redundancy. Further work will be needed to establish the role of topo VI in bacteria.

At first glance, the type II topo redundancy seen in plants can seemingly be explained by the adoption of topo VI as a highly specialized decatenase (75) that participates in the cellular process of endoreduplication (10,22,23). However, no unique DNA replication intermediates have been identified in endoreduplicating plant cells that could explain the requirement of an additional type II topo, and endoreduplication is prevalent in eukaryotes that do not possess topo VI (76). Furthermore, it is unclear why this function has not been adopted instead by topo II, or whether topo VI participates in endoreduplication in protists.

The eukaryotic topo VI complex requires two accessory proteins, RHL1 (24) and BIN4 (25), that are absent in bacteria and archaea and it is worth speculating that they play a role in further specializing topo VI and separating its activities from topo II. Interestingly, the eukaryotic dinoflagellate *C*. *goreaui*, which acquired topo VI laterally from bacteria, is an exception to this and does not seem to possess RHL1 or BIN4. It is plausible that the type II topo redundancy seen in *C*. *goreaui*, Pseudomonadota and Terrabacteria can be explained by topo VI adopting a unique or more efficient type II topo activity in these organisms, possibly by interacting with alternative accessory proteins, that significantly improves cellular fitness. It is also curious to consider whether the ‘eukaryotic’ topo VI-RHL1-BIN4 complex present in the dinoflagellate *P. donghaiense* performs an analogous role to the ‘bacterial’ topo VI of its close relative *C*. *goreaui*. However, although attempts were made to identify and remove contaminant bacterial sequences from the assembly of the *C*. *goreaui* genome (77), it remains possible that its putative topo VI is an artefact from a co-existing bacterial species.

The type II topos must stringently control the formation of DSBs to avoid deleterious DNA damage, and it is plausible that a type II topo scaffold has been adopted by the meiotic DSB machinery for its tightly regulated mechanism of DNA cleavage. However, it is unclear why eukaryotes have chosen the topo VI template, and not that of another type II topo, for meiotic recombination. The major difference between the type IIA and type IIB topos is the number of dimer interfaces, with the type IIA and type IIB enzymes possessing three and two gates, respectively (2). The simplicity of two-gate type II topo may have been a more attractive evolutionary starting point for forming a controlled DSB module, while offering an easier route for the elimination of topo activity. Topo VI-A is an ideal basic nuclease module, which unlike the type IIA enzymes, possesses the core DNA cleavage domains, toprim and WHD, in a single subunit, and has been adopted as the meiotic Spo11 with no obvious structural or sequence motif alterations. The identification of an ancient topo VI-A/Spo11 homologue in viruses, dubbed ‘Mini-A’, suggests that this nuclease module has thrived across evolutionary timelines (64). Loss of the helix-2-turn helix domain from the topo VI-B subunit and a reduction of its capacity for ATP hydrolysis seems to have been sufficient to convert the topo VI complex into an enzyme capable of performing controlled permanent DSBs without strand passage. It is unclear whether the B subunit partner of Spo11 has completely lost its capability for ATP utilization, or whether it has evolved a slower mechanism of ATP hydrolysis to regulate meiotic DSB formation. It is plausible that the B subunit of topo VI provides an important mechanistic role independent of ATP hydrolysis that has been retained by the topo VI-like complex.

A recent eukaryotic ‘Tree of Life’ has been proposed that separates nearly all major lineages into four clans: Diaphoretickes, Amorphea, CRuMs and Excavates (78). Topo VI seems to have been retained in Diaphoretickes, which is composed of Archaeplastida, Cryptista, Haptista and SAR, and to have been lost in the other three clans. Telonemia, a sister group to SAR, is the only major phylum in Diaphoretickes where topo VI has not yet been found, although this may be due to its limited available genomic data as even Spo11 has yet to be identified here. That being said, the sparse distribution of topo VI in Alveolata and Rhizaria may point instead to a small corner of Diaphoretickes where topo VI has indeed been lost. The holozoan phyla Ichthyosporea, Filasterea, and Choanoflagellatea are close animal relatives in the Amorphea group, and so their retention of topo VI is unusual and breaks the Diaphoretickes trend. A more recent phylogenetic analysis has placed the root of the eukaryotic tree within the Excavates and has identified Diaphoretickes and Amorphea as the endmost branches of eukaryotes (79). This model suggests that topo VI has been lost in most eukaryotic lineages after their divergence from the LECA, but has survived a long evolutionary path to be retained at the most recently-formed clans. Topo VI may therefore have performed an essential function in early eukaryotes that has persisted in Diaphoretickes and some holozoans but that is no longer required in other modern eukaryotes.

Topo VI is a highly specialized type II topo that is widely distributed across the three domains of life and its unique properties have deemed it a useful enzyme for adoption as the meiotic DSB machinery and for working in parallel with other type II topos. The precise nature of these properties, however, remains unclear.

## Supplementary Material

lqae085_Supplemental_File

## Data Availability

All datasets used in this study were deposited in the Figshare repository and are available under DOI: 10.6084/m9.figshare.25416106.

## Notes

Present address: Adam Allen, Department of Biochemistry, University of Cambridge, Cambridge CB2 1GA, UK.
