# Supplementary material for: Phylogenetic distribution of DNA topoisomerase VI and its distinction from SPO11
Source: NAR Genom Bioinform. 2024 Aug 6;6(3):lqae085. doi: 10.1093/nargab/lqae085 (PMC11302465; doi:10.1093/nargab/lqae085)
Supplement: lqae085_Supplemental_File [file lqae085_supplemental_file.pdf]

SUPPLEMENTARY MATERIALS

Supplementary Table 1. Distribution of collected archaeal topo VI sequences.

Organism	Superphylum	Phylum	Class	Order	Family
Freyarchaeota archaeon	Asgard	Freyarchaeota			
Heimdallarchaeota archaeon	Asgard	Heimdallarchaeota			
Helarchaeota archaeon	Asgard	Helarchaeota			
Prometheoarchaeum syntrophicum	Asgard	Lokiarchaeota	Lokiarchaeia	Prometheoarchaeales	Prometheoarchaeaceae
Odinarchaeum yellowstonii	Asgard	Odinarchaeota	Odinarchaeia	Odinarchaeales	Odinarchaeaceae
Thorarchaeota archaeon SMTZ1-45	Asgard	Thorarchaeota			
Micrarchaeum sp.	DPANN	Micrarchaeota	Micrarchaeia	Micrarchaeales	Micrarchaeaceae
Nanobsidianus stetteri	DPANN	Nanoarchaeota	Nanoarchaeia	Nanoarchaeales	Nanopusillaceae
Bathyarchaeota archaeon A05DMB-5	TACK	Bathyarchaeota			
Acidilobus saccharovorans	TACK	Crenarchaeota	Thermoprotei	Acidilobales	Acidilobaceae
Saccharolobus shibatae	TACK	Crenarchaeota	Thermoprotei	Sulfolobales	Sulfolobaceae
Pyrobaculum aerophilum	TACK	Crenarchaeota	Thermoprotei	Thermoproteales	Thermoproteaceae
Conexivisphaera calida	TACK	Nitrososphaerota	Conexivisphaeria	Conexivisphaerales	Conexivisphaeraceae
Nitrosarchaeum koreense	TACK	Nitrososphaerota	Nitrososphaeria	Nitrosopumilales	Nitrosopumilaceae
Nitrososphaera gargensis	TACK	Nitrososphaerota	Nitrososphaeria	Nitrososphaerales	Nitrososphaeraceae
Caldisphaera lagunensis	TACK	Thermoproteota	Thermoprotei	Acidilobales	Caldisphaeraceae
Ignisphaera aggregans DSM 17230	TACK	Thermoproteota	Thermoprotei	Desulfurococcales	Desulfurococcaceae
Pyrolobus fumarii	TACK	Thermoproteota	Thermoprotei	Desulfurococcales	Pyrodictiaceae
Thermofilum pendens	TACK	Thermoproteota	Thermoprotei	Thermofilales	Thermofilaceae
Geoglobus ahangari		Euryarchaeota	Archaeoglobi	Archaeoglobales	Archaeoglobaceae
Halomicroarcula salinisoli		Euryarchaeota	Halobacteria	Halobacteriales	Haloarculaceae
Halobacterium salinarum		Euryarchaeota	Halobacteria	Halobacteriales	Halobacteriaceae
Halopelagius inordinatus		Euryarchaeota	Halobacteria	Haloferacales	Haloferacaceae
Salinigranum halophilum		Euryarchaeota	Halobacteria	Haloferacales	Halorubraceae
Natronorubrum sediminis		Euryarchaeota	Halobacteria	Natrialbales	Natrialbaceae
Methanothermobacter defluvii		Euryarchaeota	Methanobacteria	Methanobacteriales	Methanobacteriaceae
Methanothermus fervidus		Euryarchaeota	Methanobacteria	Methanobacteriales	Methanothermaceae
Methanocaldococcus jannaschii		Euryarchaeota	Methanococci	Methanococcales	Methanocaldococcaceae
Methanococcus voltae		Euryarchaeota	Methanococci	Methanococcales	Methanococcaceae
Methanosarcina mazei		Euryarchaeota	Methanomicrobia	Methanosarcinales	Methanosarcinaceae
Thermococcus kodakarensis		Euryarchaeota	Thermococci	Thermococcales	Thermococcaceae
Methanomassiliicoccaceae archaeon DOK		Euryarchaeota	Thermoplasmata	Methanomassiliicoccales	Methanomassiliicoccaceae
Cuniculiplasma divulgatum		Euryarchaeota	Thermoplasmata	Thermoplasmatales	Cuniculiplasmataceae
Thermogymnomonas acidicola[a]		Euryarchaeota	Thermoplasmata	Thermoplasmatales	*Uncharacterised*

[a]Topo VI sequences from *Thermogymnomonas acidicola* were not included in the phylogenetic analysis

Supplementary Table 2. Distribution of collected bacterial topo VI sequences

Organism	Superphylum	Phylum	Class	Order	Family
Anaerohalosphaera lusitana	PVC	Planctomycetota	Phycisphaerae	Sedimentisphaerales	Anaerohalosphaeraceae
Sedimentisphaera cyanobacteriorum	PVC	Planctomycetota	Phycisphaerae	Sedimentisphaerales	Sedimentisphaeraceae
Lacipirellula parvula	PVC	Planctomycetota	Planctomycetia	Pirellulales	Lacipirellulaceae
Pirellula staleyi	PVC	Planctomycetota	Planctomycetia	Pirellulales	Pirellulaceae
Thermogutta terrifontis	PVC	Planctomycetota	Planctomycetia	Pirellulales	Thermoguttaceae
Ferrimicrobium acidiphilum	Terrabacteria	Actinomycetota	Acidimicrobiia	Acidimicrobiales	Acidimicrobiaceae
Cyanobacterium TDX16	Terrabacteria	Cyanobacteriota			
Bdellovibrio bacteriovorus		Bdellovibrionota	Bdellovibrionia	Bdellovibrionales	Pseudobdellovibrionaceae
Anaeromyxobacter dehalogenans		Myxococcota	Myxococcia	Myxococcales	Anaeromyxobacteraceae
Vulgatibacter incomptus		Myxococcota	Myxococcia	Myxococcales	Vulgatibacteraceae
Pajaroellobacter abortibovis		Myxococcota	Polyangia	Polyangiales	Polyangiaceae
Nitrospira moscoviensis		Nitrospirae	Nitrospira	Nitrospirales	Nitrospiraceae
Methylocystis sp. NLS-7		Pseudomonadota	Alphaproteobacteria	Hyphomicrobiales	Methylocystaceae
Bradyrhizobium sediminis		Pseudomonadota	Alphaproteobacteria	Hyphomicrobiales	Nitrobacteraceae
Roseibium aggregatum		Pseudomonadota	Alphaproteobacteria	Hyphomicrobiales	Stappiaceae
Marimonas lutisalis		Pseudomonadota	Alphaproteobacteria	Rhodobacterales	Paracoccaceae
Actibacterium sp. MT2.3-13A		Pseudomonadota	Alphaproteobacteria	Rhodobacterales	Roseobacteraceae

Supplementary Table 3. Distribution of collected eukaryotic topo VI sequences

Organism	Supergroup	Phylum	Class	Order	Family
Chlamydomonas reinhardtii	Archaeplastida	Chlorophyta	Chlorophyceae	Chlamydomonadales	Chlamydomonadaceae
Tetradesmus obliquus	Archaeplastida	Chlorophyta	Chlorophyceae	Sphaeropleales	Scenedesmaceae
Chloropicon primus	Archaeplastida	Chlorophyta	Chloropicophyceae	Chloropicales	Chloropicaceae
Ostreococcus tauri	Archaeplastida	Chlorophyta	Mamiellophyceae	Mamiellales	Bathycoccaceae
Micromonas commoda	Archaeplastida	Chlorophyta	Mamiellophyceae	Mamiellales	Mamiellaceae
Micractinium conductrix	Archaeplastida	Chlorophyta	Trebouxiophyceae	Chlorellales	Chlorellaceae
Coccomyxa subellipsoidea	Archaeplastida	Chlorophyta	Trebouxiophyceae	Chlorococcales	Coccomyxaceae
Cyanophora paradoxa	Archaeplastida	Glaucophyta	Glaucophyceae	Glaucocystales	Cyanophoraceae
Gloeochaete wittrockiana	Archaeplastida	Glaucophyta	Glaucophyceae	Gloeochaetales	Gloeochaetaceae
Cyanidioschyzon merolae strain 10D	Archaeplastida	Rhodophyta	Bangiophyceae	Cyanidiales	Cyanidiaceae
Porphyridium purpureum	Archaeplastida	Rhodophyta	Bangiophyceae	Porphyridiales	Porphyridiaceae
Chondrus crispus	Archaeplastida	Rhodophyta	Florideophyceae	Gigartinales	Gigartinaceae
Gracilariopsis chorda	Archaeplastida	Rhodophyta	Florideophyceae	Gracilariales	Gracilariaceae
Physcomitrella patens	Archaeplastida	Streptophyta	Bryopsida	Funariales	Funariaceae
Klebsormidium nitens	Archaeplastida	Streptophyta	Klebsormidiophyceae	Klebsormidiales	Klebsormidiaceae
Selaginella moellendorffii	Archaeplastida	Streptophyta	Lycopodiopsida	Selaginellales	Selaginellaceae
Amborella trichopoda	Archaeplastida	Streptophyta	Magnoliopsida	Amborellales	Amborellaceae
Arabidopsis thaliana	Archaeplastida	Streptophyta	Magnoliopsida	Brassicales	Brassicaceae
Spinacia oleracea	Archaeplastida	Streptophyta	Magnoliopsida	Caryophyllales	Chenopodiaceae
Juglans regia	Archaeplastida	Streptophyta	Magnoliopsida	Fagales	Juglandaceae
Coffea canephora	Archaeplastida	Streptophyta	Magnoliopsida	Gentianales	Rubiaceae
Cinnamomum micranthum	Archaeplastida	Streptophyta	Magnoliopsida	Laurales	Lauraceae
Populus trichocarpa	Archaeplastida	Streptophyta	Magnoliopsida	Malpighiales	Salicaceae
Punica granatum	Archaeplastida	Streptophyta	Magnoliopsida	Myrtales	Lythraceae
Oryza sativa	Archaeplastida	Streptophyta	Magnoliopsida	Poales	Poaceae
Aquilegia coerulea	Archaeplastida	Streptophyta	Magnoliopsida	Ranunculales	Ranunculaceae
Prunus dulcis	Archaeplastida	Streptophyta	Magnoliopsida	Rosales	Rosaceae
Citrus unshiu	Archaeplastida	Streptophyta	Magnoliopsida	Sapindales	Rutaceae
Nicotiana attenuata	Archaeplastida	Streptophyta	Magnoliopsida	Solanales	Solanaceae
Vitis vinifera	Archaeplastida	Streptophyta	Magnoliopsida	Vitales	Vitaceae
Marchantia polymorpha	Archaeplastida	Streptophyta	Marchantiopsida	Marchantiales	Marchantiaceae
Cryptomeria japonica	Archaeplastida	Streptophyta	Pinopsida	Cupressales	Cupressaceae
Guillardia theta	Cryptista	Cryptophyta	Cryptophyceae	Pyrenomonadales	Geminigeraceae
Palpitomonas bilix	Cryptista	Cryptophyta	Palpitea	Palpitida	Palpitomonadidae
Emiliania huxleyi	Haptista	Haptophyta	Prymnesiophyceae	Isochrysidales	Noelaerhabdaceae
Phaeocystis globosa	Haptista	Haptophyta	Prymnesiophyceae	Phaeocystales	Phaeocystaceae
Chrysochromulina tobinii	Haptista	Haptophyta	Prymnesiophyceae	Prymnesiales	Chrysochromulinaceae
Monosiga brevicollis	Opisthokonta	Choanozoa	Choanoflagellatea	Craspedida	Salpingoecidae
Capsaspora owczarzaki	Opisthokonta	Choanozoa	Filasterea	Ministeriida	Capsasporidae
Amoebidium parasiticum	Opisthokonta	Choanozoa	Ichthyosporea	Ichthyophonida	Amoebidiaceae
Sphaeroforma arctica	Opisthokonta	Choanozoa	Ichthyosporea	Ichthyophonida	Creolimacidae
Cafeteria roenbergensis	SAR	Bigyra	Bikosea	Bicosoecida	Cafeteriaceae
Hondaea fermentalgiana	SAR	Bigyra	Labyrinthulea	Thraustochytrida	Thraustochytriaceae
Lotharella oceanica	SAR	Cercozoa	Chlorarachniophyceae	Chlorarachniales	Chlorarachniaceae
Prorocentrum donghaiense	SAR	Myzozoa	Dinophyceae	Prorocentrales	Prorocentraceae
Cladocopium goreaui	SAR	Myzozoa	Dinophyceae	Suessiales	Symbiodiniaceae
Mayamaea pseudoterrestris	SAR	Ochrophyta	Bacillariophycea	Naviculales	Naviculaceae
Fragilariopsis cylindrus	SAR	Ochrophyta	Bacillariophyceae	Bacillariales	Bacillariaceae
Phaeodactylum tricornutum CCAP 1055/1	SAR	Ochrophyta	Bacillariophyceae	Naviculales	Phaeodactylaceae
Tetraparma gracilis	SAR	Ochrophyta	Bolidophyceae	Parmales	Triparmaceae
Chaetoceros tenuissimus	SAR	Ochrophyta	Coscinodiscophyceae	Chaetocerotales	Chaetocerotaceae
Thalassiosira pseudonana	SAR	Ochrophyta	Coscinodiscophyceae	Thalassiosirales	Thalassiosiraceae
Nannochloropsis gaditana	SAR	Ochrophyta	Eustigmatophyceae	Eustigmatales	Monodopsidaceae
Pelagomonas calceolata	SAR	Ochrophyta	Pelagophyceae	Pelagomonadales	Pelagomonadaceae
Ectocarpus siliculosus	SAR	Ochrophyta	Phaeophyceae	Ectocarpales	Ectocarpaceae
Saccharina japonica	SAR	Ochrophyta	Phaeophyceae	Laminariales	Laminariaceae
Pythium insidiosum	SAR	Oomycota	Oomycetes	Pythiales	Pythiaceae
Aphanomyces astaci	SAR	Oomycota	Oomycetes	Saprolegniales	Saprolegniaceae

Supplementary Table 4. Distribution of collected Apicomplexa SPO11 and pTOP6B sequences

Class	Order	Family	Genus	SPO11	pTOP6B
Aconoidasida	Haemosporida	Plasmodiidae	Plasmodium	2	1
	Piroplasmida	Babesiidae	Babesia	2	1
		Theileriidae	Theileria	2	1
Conoidasida	Eucoccidiorida	Cryptosporidiidae	Cryptosporidium	2	1
		Eimeriidae	Cyclospora	2	1
		Sarcocystidae	Toxoplasma	2	1
	Eugregarinorida	Porosporidae	Porospora	2	1

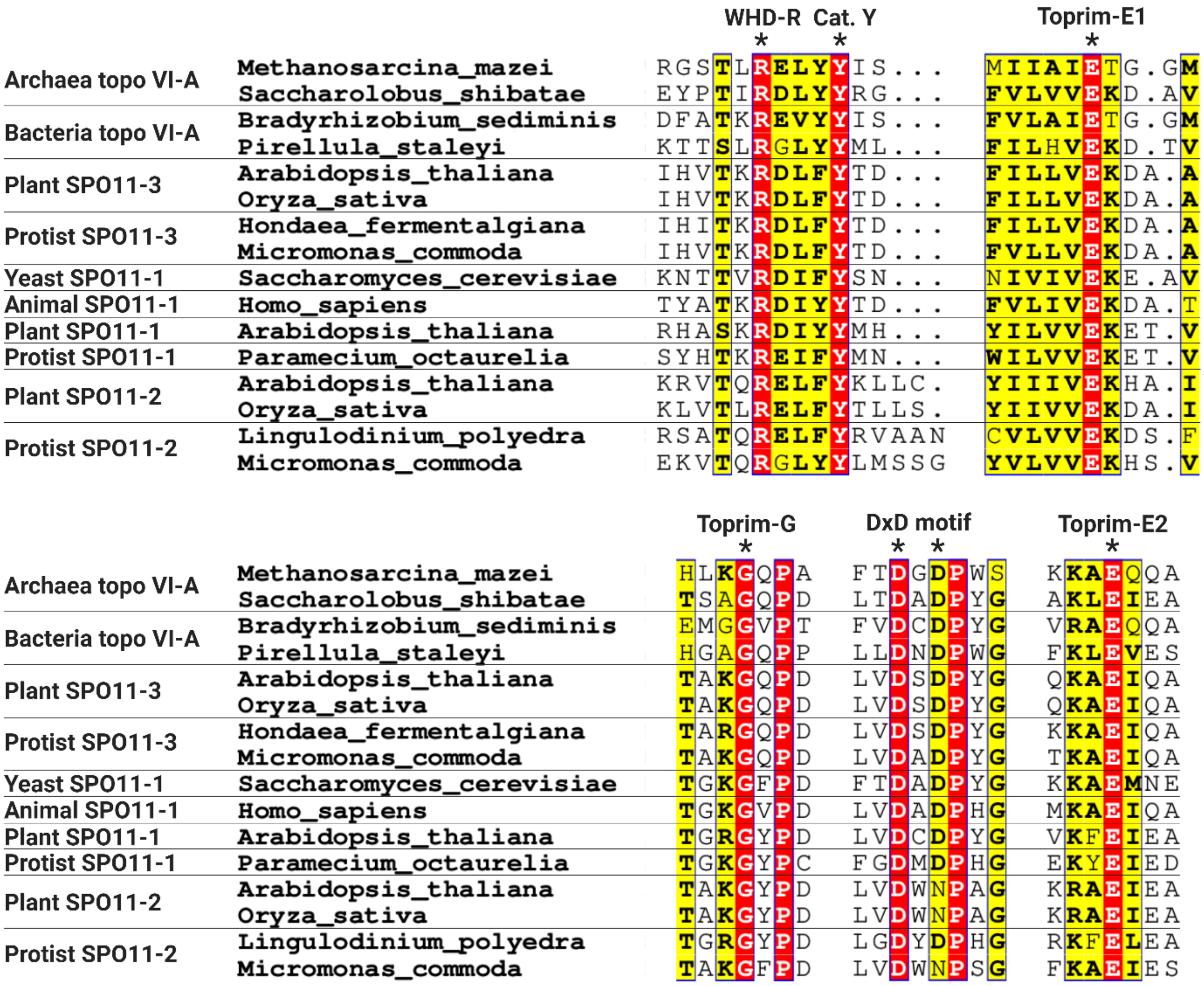

Supplementary Figure 1. Sequence alignment of the topo VI-A/Spo11 family: All members of the topo VI-A/Spo11 family contain key invariant residues in the winged-helix domain (WHD) and toprim domain. The WHD possess the catalytic tyrosine (Cat. Y) and an adjacent arginine (WHD-R). Toprim domains are characterised by a conserved glutamate (toprim-E1) and two conserved aspartates (DxD) which coordinate the essential divalent metal ion. Topo VI homologues also contain a conserved glutamate (toprim-E2) in the active site that hydrogen bonds with the WHD-R and Cat. Y and contain a conserved glycine (toprim-G) that sits in the DNA-binding pocket. In the SPO11-2 subfamily, the second aspartate residue in the DxD motif is often substituted for asparagine.

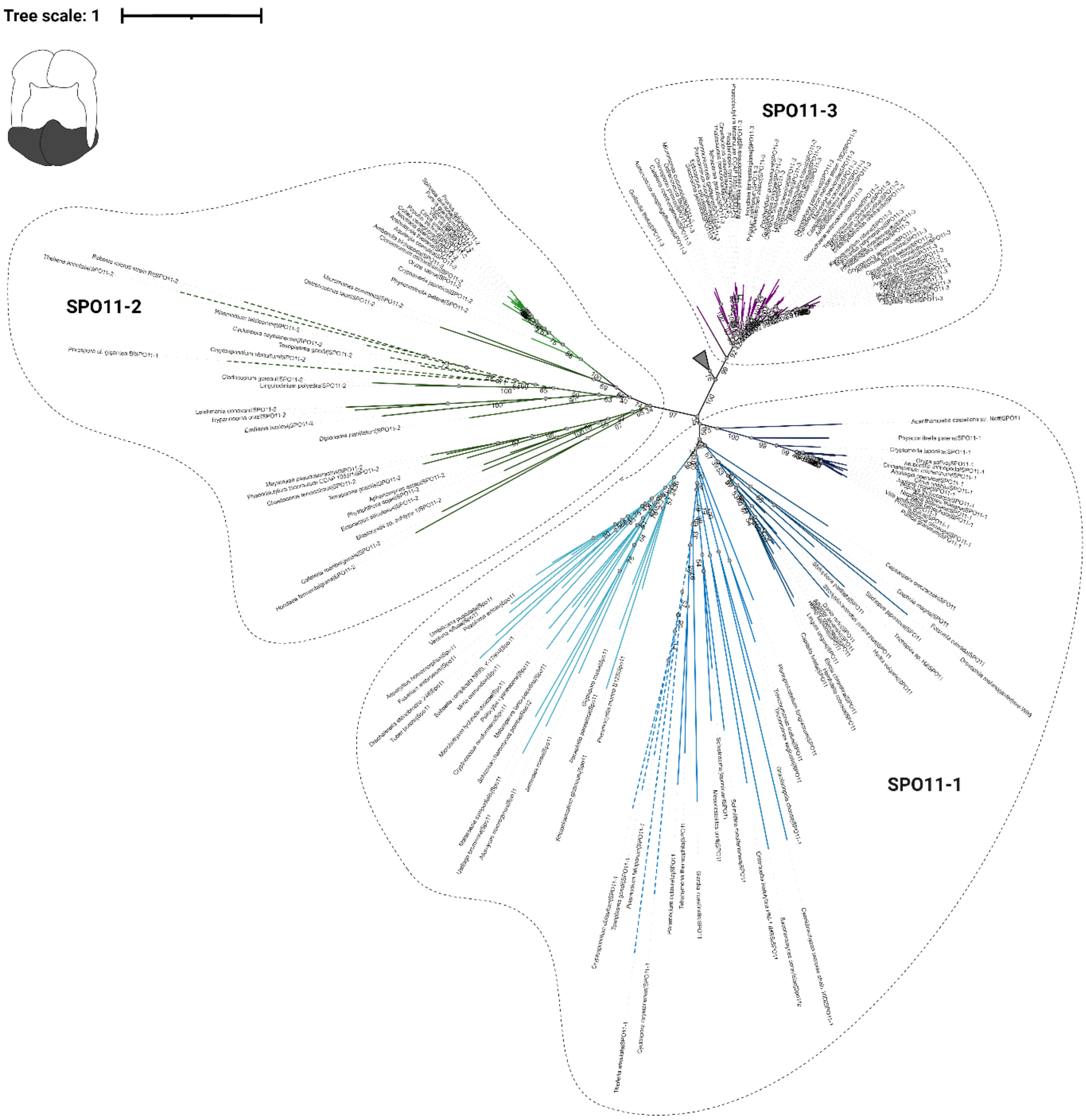

Supplementary Figure 2. Phylogenetic tree of the eukaryotic Spo11 family: An unrooted maximum-likelihood tree using 223 aligned topo VI-A/Spo11 sequences including 33 archaea, 17 bacteria, and 173 eukaryotes. The eukaryotic Spo11 family splits into three major clades: SPO11-1, SPO11-2, and SPO11-3, and the prokaryotic node has been collapsed and is displayed as a triangle. The dotted leaves represent Apicomplexa SPO11 paralogues and the cartoon in the top left corner depicts the structure of topo VI (15) with the A subunit shaded in black. Tree was generated from a trimmed alignment possessing 263 amino acids and the tree scale represents the number of amino acid substitutions per site. Bootstrap values are displayed at the midpoint of branches and internal nodes are displayed as circles.

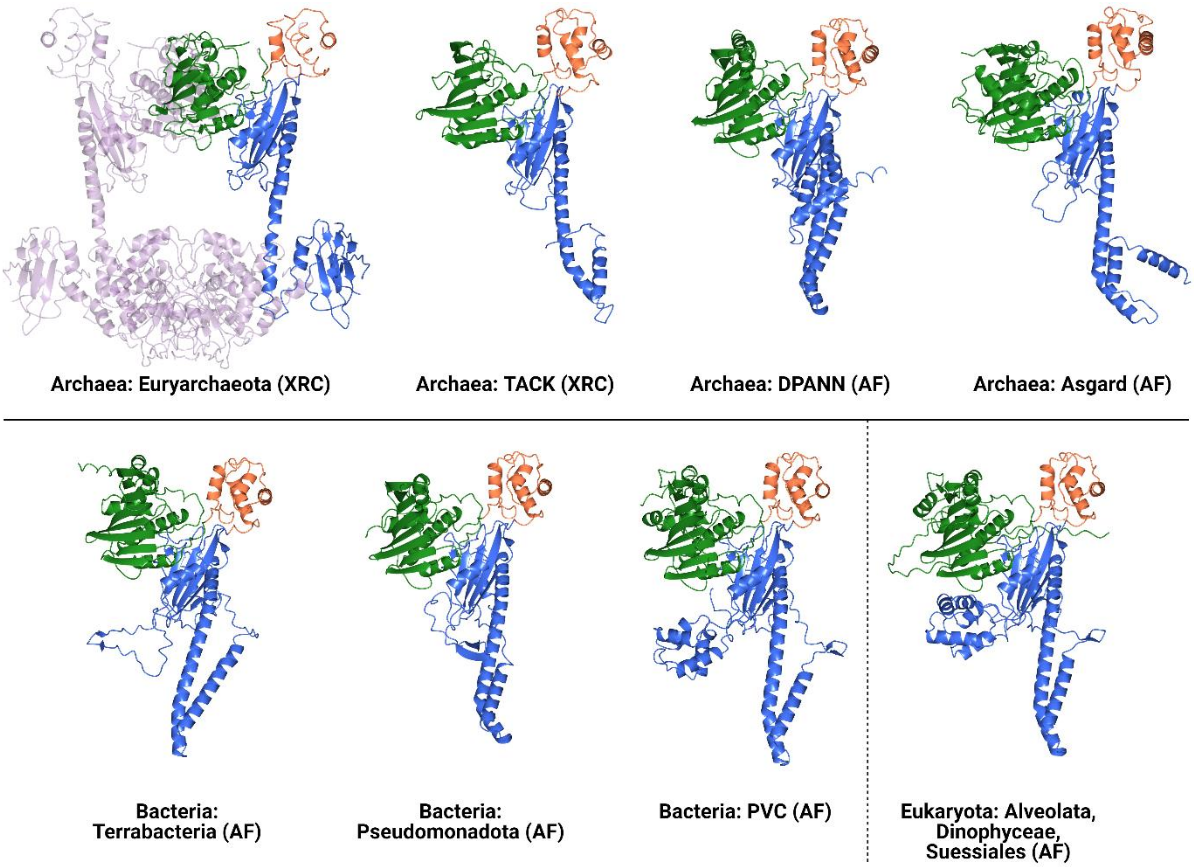

Supplementary Figure 3. Prokaryotic topo VI-B structural modelling: The X-ray crystal (XRC) structure of *Methanosarcina mazei* (Euryarchaeota) topo VI (PDB code: 2Q2E) is a heterotetramer formed of two topo VI-As and two topo VI-Bs. The *M. mazei* topo VI-B structure is comparable to the *Saccharolobus shibatae* (TACK) topo VI-B XRC structure (PDB code: 2ZBK), which both feature a transducer domain (blue), a H2TH domain (orange), and a GHKL domain (green). AlphaFold (AF) structural modelling predicts that the topo VI-B structure is also conserved in DPANN (*Candidatus Nanobsidianus stetteri*) and Asgard (*Candidatus Odinarchaeum yellowstonii*) archaea; and Terrabacteria (*Cyanobacterium TDX16*), Pseudomonodata (*Bradyrhizobium sediminis*), and PVC (*Pirellula staleyi*) bacteria. Topo VI-B from the eukaryote *Cladocopium goreaui* (Suessiales) was acquired from a PVC bacterium by horizontal gene transfer and shares a common alpha-helical bundle in its transducer domain.

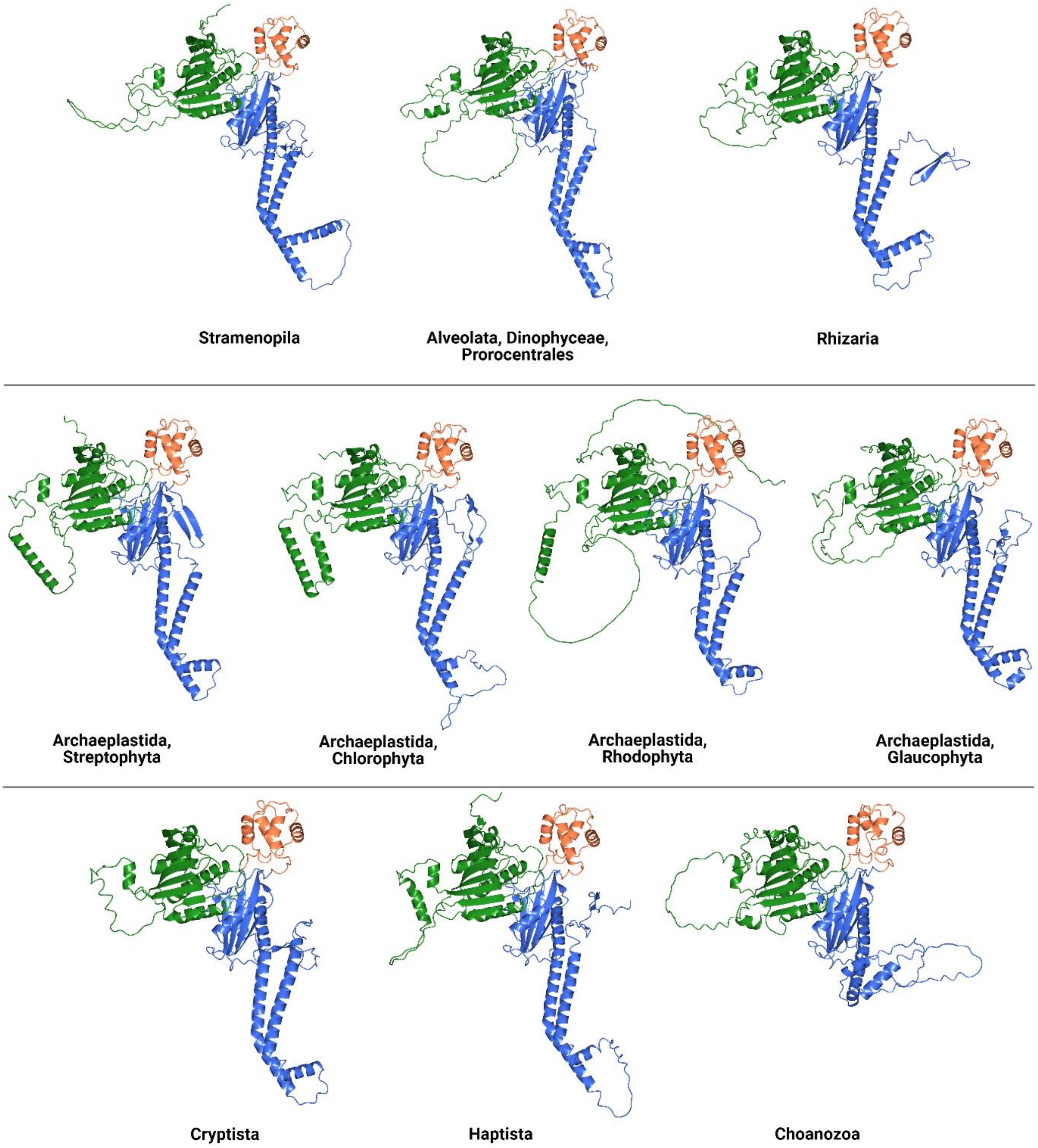

Supplementary Figure 4. Eukaryotic topo VI-B structural modelling: AlphaFold structural modelling predicts that the topo VI-B structure, featuring a transducer domain (blue), a H2TH domain (orange), and a GHKL domain (green), is conserved in Streptophyta (*Arabidopsis thaliana*), Chlorophyta (*Chlamydomonas reinhardtii*), Rhodophyta (*Chondrus crispus*), Glaucophyta (*Cyanophora paradoxa*), Cryptista (*Guillardia theta*), Haptista (*Phaeocystis globosa*), and Choanozoa (*Monosiga brevicollis*).

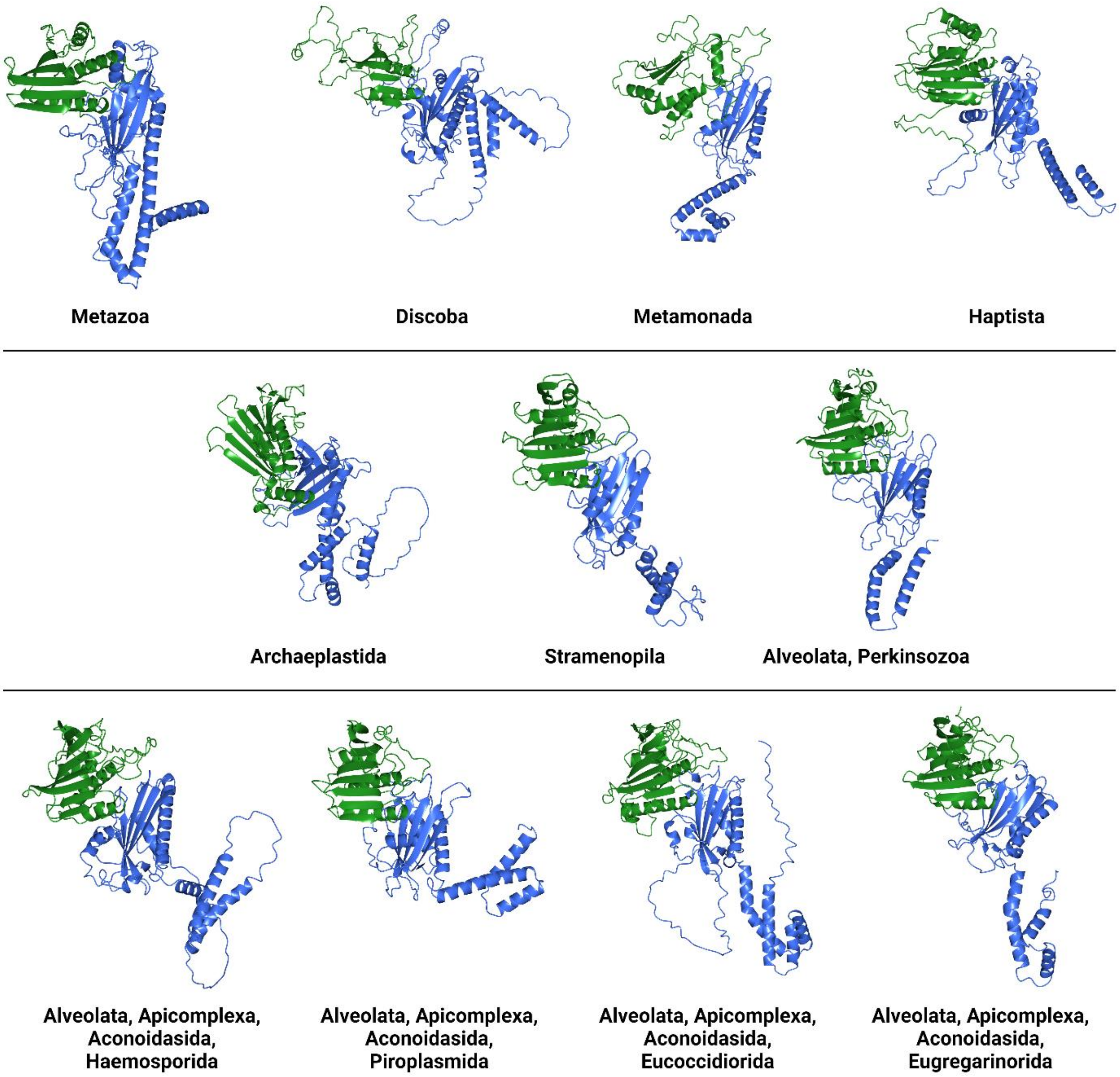

Supplementary Figure 5. Topo VIB-like structural modelling: AlphaFold structural modelling predicts that the topo VIB-like family possess a transducer-like domain (blue) and a GHKL-like domain (green), but do not possess a H2TH domain. These structures are represented by Metazoa (*Homo sapiens*), Discoba (*Trypanosoma theileri*), Metamonada (*Giardia intestinalis*), Haptista (*Emiliania huxleyi*), Archaeplastida (*Arabidopsis thaliana*), Stramenopila (*Aphanomyces euteiches*), Perkinsozoa (*Perkinsus chesapeaki*), Haemosporida (*Plasmodium falciparum*), Piroplasmida (*Babesia microti*), Eucoccidiorida (*Cryptosporidium ubiquitum*), and Eugregarinorida (*Porospora cf. gigantea B*).

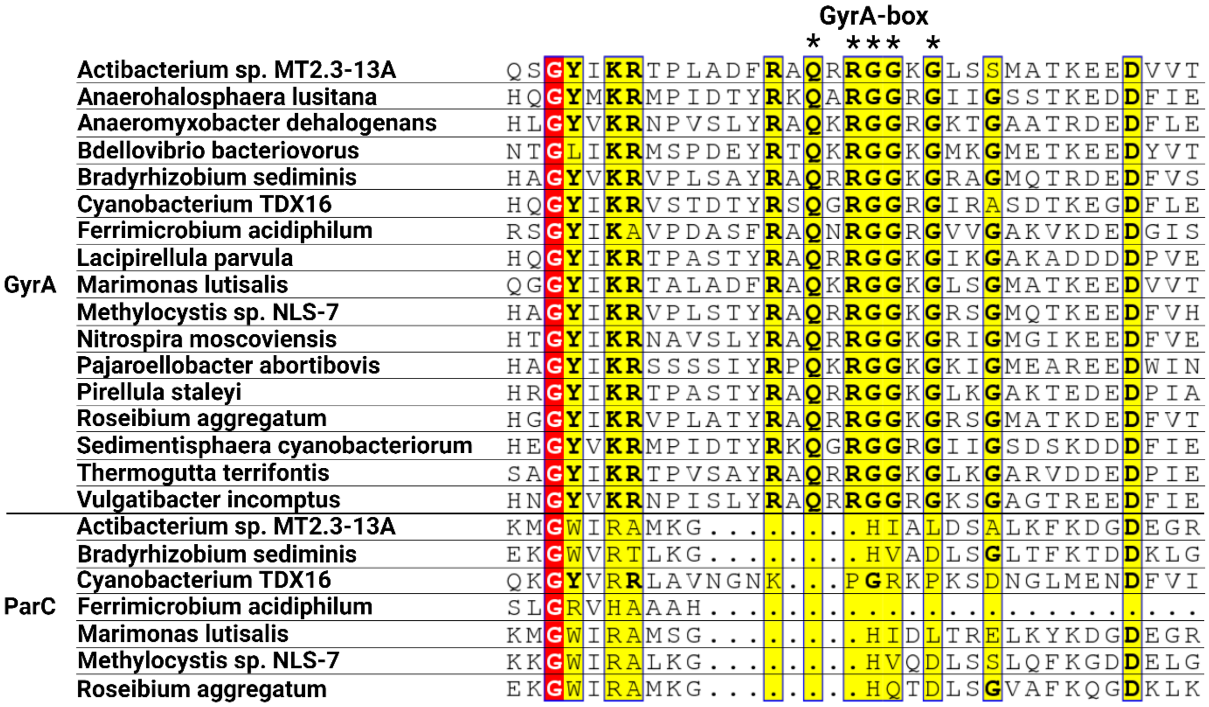

Supplementary Figure 6. Sequence alignment of the bacterial GyrA-box: Putative GyrA/ParC sequences of topo VI-possessing bacteria from 17 unique families were aligned at the GyrA-box region. All topo VI-possessing species possess GyrA, and seven also possess ParC.
